# Cholinesterase Depression and Its Association with Pesticide Exposure across the Agricultural Season among Latino Farmworkers in North Carolina

**DOI:** 10.1289/ehp.0901492

**Published:** 2010-01-19

**Authors:** Sara A. Quandt, Haiying Chen, Joseph G. Grzywacz, Quirina M. Vallejos, Leonardo Galvan, Thomas A. Arcury

**Affiliations:** 1 Department of Epidemiology and Prevention; 2 Department of Biostatistical Sciences, Division of Public Health Sciences and; 3 Department of Family and Community Medicine, Wake Forest University School of Medicine, Winston-Salem, North Carolina, USA; 4 North Carolina Farmworkers Project, Benson, North Carolina, USA

**Keywords:** cholinesterase, farmworker, pesticide

## Abstract

**Background:**

Farmworkers can be exposed to a wide variety of pesticides. Assessing cholinesterase activity over time can be used to monitor exposure to organophosphorus and carbamate pesticides.

**Objectives:**

The goal of this study was to document patterns and variation in cholinesterase levels across the agricultural season (May–August) among field-workers, and to explore the association of cholinesterase depression with pesticide exposure across the agricultural season.

**Methods:**

Dried blood samples collected from 231 migrant farmworkers sampled from camps in eastern North Carolina up to four times across a summer agricultural season were analyzed for cholinesterase activity, and urine samples were analyzed for metabolites of organophosphorus and carbamate pesticides. Reductions of ≥ 15% from an individual’s highest value were identified and considered evidence of meaningful cholinesterase activity depression.

**Results:**

The average cholinesterase activity levels were lowest in June, with significantly higher mean values in July and August. When adjusted for age, sex, minutes waited to shower, and days worked in the fields, the number of organophosphorus and carbamate pesticides detected in urine predicted reductions in cholinesterase activity.

**Conclusions:**

These data demonstrate that workers are experiencing pesticide exposure. Greater enforcement of existing safety regulations or strengthening of these regulations may be warranted. This study demonstrates that serial measurements of cholinesterase activity across an agricultural season can detect exposure to pesticides among field-workers.

Farmworkers can be exposed to a variety of pesticides in their work. Although educational programs such as those based on the U.S. Environmental Protection Agency (EPA) Worker Protection Standard promote preventive behaviors, including the use of personal protective equipment and hygiene, studies indicate that exposure occurs for a significant proportion of workers and their coresident family members (Arcury et al. 2009a, 2009b; Bradman et al. 2007; Hernández-Valero et al. 2001). Monitoring cholinesterase activity provides a means of assessing exposure to organophosphorus and carbamate pesticides (Nigg and Knaak 2000). These pesticides inhibit acetylcholinesterase activity (AChE; Enzyme Commission number EC 3.1.1.2) and may produce an array of neurotoxic effects (reviewed in Gupta 2006). Because of the essentially irreversible binding of organophosphorus pesticides to red blood cell cholinesterase, recovery from cholinesterase depression is prolonged, reflecting the 120-day half-life of red blood cells. This, plus the wide range of normal values for cholinesterase, makes repeated measures the best means of detecting cholinesterase depression (Flegar-Meštrić et al. 1999; Lessenger 2005; Lessenger and Reese 1999; Mutch et al. 1992). Routine screening for cholinesterase depression is mandated in only a few places [e.g., California and Washington State (Hofmann et al. 2008; Lessenger 2005)] and then only for workers applying and handling pesticides and not those carrying out routine fieldwork.

Most research on cholinesterase in farm laborers focuses on applicators or workers exposed to high concentrations of pesticides, often in developing countries, through misuse (Gamlin et al. 2007; Jørs et al. 2006; Khan et al. 2009; Lu 2007). Although it has been hypothesized that long-term exposure to levels of pesticides too low to cause symptoms of poisoning would also be detectable through cholinesterase sampling, few published studies have used repeated sampling of cholinesterase to document such low-level exposure in workers.

In this article we focus on data collected from Latino farmworkers in North Carolina across an agricultural season. We used data on cholinesterase activity obtained from whole blood samples, pesticide dose data obtained from urine samples, and behavioral data concerning work activities obtained from self-reports. Our goals were to *a*) document patterns and variation in cholinesterase levels across the agricultural season and *b*) explore the association of cholinesterase depression with pesticide exposure across the agricultural season.

## Methods

Community Participatory Approach to Measuring Farmworker Pesticide Exposure (PACE3) is an ongoing translational research program addressing the health of Latino farmworkers in eastern North Carolina. PACE3 used a longitudinal design in which data were collected from participants up to four times at monthly intervals in 2007. All sampling, recruitment, and data collection protocols, including signed informed consent, were approved by the Wake Forest University School of Medicine Institutional Review Board, in compliance with all applicable U.S. requirements. Informed consent was obtained from each participant before any study data were collected.

### Locale

Data collection was completed during the summer of 2007 in 11 counties in North Carolina with large farmworker populations: Brunswick, Columbus, Cumberland, Greene, Harnett, Johnston, Lenoir, Pitt, Sampson, Wayne, and Wilson counties. Conservative estimates by the North Carolina Employment Security Commission (unpublished data) in 2007 put the number of migrant farmworkers in these counties without H-2A (guest-worker) visas at 13,675 and with H-2A visas at 2,995, and the number of seasonal farmworkers at 5,800. The workers in these counties constitute substantial proportions of the total farmworker population in North Carolina, an estimated 36.2% of migrant workers without H-2A visas, 34.3% of migrant farmworkers with H-2A visas, and 22.8% of seasonal workers. The agricultural production in these counties varies, but the major hand-cultivated and hand-harvested crops include tobacco, sweet potatoes, and cucumbers.

### Sample

PACE3 used a two-stage procedure to select farmworkers. Details are presented elsewhere (Arcury et al. 2009a). Briefly, three partnering agencies prepared lists of farmworker camps for the counties that they served. Camps were randomized and approached in order until each agency recruited a minimum number of camps and a specified number of participants. All 44 camps that were approached agreed to participate. In camps with seven or fewer residents, all farmworkers were invited to participate. In camps with more than seven residents, 8–10 farmworkers were recruited. In total, 287 farmworkers were recruited: 261 at the first round of data collection, and 26 at the second round of data collection. Of all farmworkers approached by the interviewers, 13 chose not to participate, for a participation rate of 95.7%. At the second round of data collection, 41 participants were lost to follow-up; 20 were lost at the third round, and 12 were lost at the fourth round. Four rounds of data collection were completed with 197 farmworkers, only three rounds with 27, only two rounds with 14, and only one round with 49.

### Data collection

Data collection relevant to these analyses included a detailed interview, a finger-stick blood sample to measure cholinesterase, and a first morning urine void to measure pesticide metabolites. Participants were given an incentive valued at $20 when they completed data collection for each round.

Data collection was completed from May through September 2007 by teams of data collectors who were fluent in Spanish. A detailed interview was completed with the farmworker participants at each round of data collection. At every contact, the questionnaire included items on work conditions in the 3 days before the interview and risk factors for pesticide exposure. At the first contact, the questionnaire also included items on participant personal characteristics (e.g., age, educational attainment) and current health status. The questionnaire used in these interviews was developed in English, translated by an experienced translator who was a native Spanish speaker familiar with Mexican Spanish, reviewed by four fluent Spanish speakers familiar with farmwork, and then pretested with 16 Spanish-speaking farmworkers and revised as needed.

Blood samples to measure cholinesterase activity were collected at each of the four interviews. The sample collector first cleaned one finger of the farmworker well with an alcohol wipe and then pricked the finger using a sterile lancet. The resulting blood drops were applied to 903 Protein Saver paper (Whatman Ltd., Piscataway, NJ), soaking through a printed half-inch circle that holds 75–80 μL blood. Samples were labeled, allowed to dry, placed in a paper envelope, and then sealed in a plastic bag for transport to the laboratory. Random duplicates were also collected and sent to the laboratory for analysis. Samples were labeled so that the laboratory could not identify individuals and duplicates.

The dried blood samples were delivered to the National Health and Environmental Effects Research Laboratory of the U.S. EPA’s Cellular and Molecular Toxicology Branch, Neurotoxicology Division (Research Triangle Park, NC), and analyzed as previously described by Hilborn and Padilla (2004). Dried blood spots were punched out of the filter paper with a standard office hole-punch. Only those that were 98–100% saturated with blood on both sides of the filter paper were used for the final analysis. Previous work had estimated that each punch contained approximately 15 μL of fresh blood. The punch was then added to 500 μL Triton/Ellman buffer (0.1 mM sodium phosphate buffer, pH 8.0, plus 1% Triton X-100). To allow the whole blood to elute, each vial containing buffer and punch was placed in a refrigerator for 17 hr and then in a shaking water bath (26°C) for an additional 4 hr. The Triton/Ellman buffer was then removed from the vial and analyzed for cholinesterase activity. Cholinesterase activity was assessed in 15 μL of each sample using a basic Ellman assay (Ellman et al. 1961) modified for use in a microtiter plate reader (Nostrandt et al. 1993). All samples from a participant were analyzed on the same day. A reference sample (rat brain homogenate) was run on each plate to ensure consistency among plates; the coefficient of variation for these reference samples was 5%. Activity is reported as nanomoles of acetylthiocholine hydrolyzed per minute per milliliter of whole blood.

For the measurement of pesticide urinary metabolites, at the end of each interview the interviewer gave the participants urine collection containers with labels attached. Participants were instructed to fill the containers with their first void upon rising the next morning. They were assured that the urine samples would be tested for agricultural chemicals and metals only and not for the use of alcohol, drugs, or any health conditions. They were asked specifically to provide only their own urine in the containers, not that of any other workers in the camp. Participants placed their urine containers in a cooler with blue ice that was provided to them, and these coolers were retrieved by project staff. Coolers were transported to the nearest of the three collaborating community partners, and samples were aliquoted into labeled containers and placed in a laboratory freezer, where they were stored at −20°C. The urine samples were shipped on dry ice to the Centers for Disease Control and Prevention (Atlanta, GA) via overnight delivery. These samples were analyzed for pesticide metabolites as described elsewhere (Arcury et al. 2009a). Metabolites included those for organophosphorus insecticides (parent chemicals: acephate, chlorpyrifos and chlorpyrifos methyl, coumaphos, diazinon, isazophos, malathion, parathion and methyl parathion, pirimiphos methyl, *o*-methoate, dimethoate), carbamates (parent chemicals: bis-dithiocarbamates), pyrethroid insecticides, and herbicides.

### Measures

To define a depression of cholinesterase in a situation where no baseline cholinesterase values were available, the percent change from an individual’s maximum value was calculated at each time point. Participants’ maximum values were assumed to be the most proximal indicator of cholinesterase recovery. Possible cut points of 10%, 15%, and 20% reductions from each individual’s maximum value were explored. A 15% reduction was considered a significant change, because it approximates the 20% reduction from baseline used by Washington State to trigger workplace and worker inquiries to address the problem, and because a true “baseline” or period of nonexposure was not available (Washington State Department of Labor and Industries 2003). Further, Lefkowitz et al. (2007) recently observed that differences of 12.1% in red blood cell cholinesterase activity have a 95% probability of being significant departures from baseline, and Garabrant et al. (2009) suggest that defining cholinesterase depression in terms of a 20% change results in a large number of false positives. We compared a total of 564 maximum values with another value for the same individual. Approximately half of the comparisons were ≥ 15%.

Data collected were divided into four periods roughly corresponding to months. May included data collected from 1 May to 8 June; June, from 9 June to 7 July; July, from 8 July to 5 August; and August, from 6 August to 4 September.

The number of pesticides detected ranged from 0 to 7, calculated at each data collection time point from the number of organophosphorus (acephate, chlorpyrifos, dimethoate, malathion, methamidophos, pirimiphos methyl) or carbamate (ethylene thiourea) pesticides with values exceeding the limit of detection.

Time waited to shower after returning home (in minutes) was reported by the worker for the most recent day worked before data collection. In most cases, this was the day of the data collection.

Age was treated as a continuous variable.

Farmworkers were coded as working 0, 1, 2, or 3 of the 3 days preceding the data collection. The 3-day look-back period corresponds with the period in which most pesticides are metabolized.

### Data analysis

Duplicate samples from 56 randomly selected individual farmworkers were collected on the same day to assess the reproducibility of cholinesterase measurements. The distribution of cholinesterase concentrations appeared to be normal. We used a mixed-model approach to estimate the within-subject SD. The resulting coefficient of variation for the duplicate samples was 10.4%.

For the main analyses of 231 farmworkers, we first examined the pattern of change in average cholinesterase levels over time. To account for the clustered longitudinal design, we fitted a mixed-effects model that explicitly modeled the correlation of repeated measures for each individual farmworker and the correlation attributed to multiple farmworkers within the same camp. Least-square means were reported for each time period, and Tukey’s method was used to evaluate pairwise differences while adjusting for multiple comparisons. Descriptive statistics, frequency counts, and percentages are presented for a depression of ≥ 15% from reference levels.

Finally, a regression model was fit to identify potential predictors for the change in cholinesterase activity. We used the difference in cholinesterase between a current month and its previous month (current – previous) as the outcome *Y* to delineate whether recent pesticide exposure is associated with contemporaneous change in cholinesterase activity. We adopted an autoregression approach for our analysis. That is, the outcome at time *t* (or *Y_t_*) was regressed on the outcome at *t* − 1 (or *Y_t_*_−1_). The model also included the total number of detects in pesticides in the current and the previous months, and change in cholinesterase from month to month (June vs. May, July vs. June, and August vs. July). Month-to-month change in cholinesterase was included as farmworkers’ endogenous variation in cholinesterase to more clearly delineate the potential effects of pesticide exposure. Multivariate models also adjusted for the maximum cholinesterase level and personal characteristics. All analyses were performed using SAS (version 9.2; SAS Institute Inc., Cary, NC), and *p*-value < 0.05 was considered statistically significant.

## Results

Six individuals were removed from the analysis because of missing data due to a laboratory error (2 observations) and blood spots with inadequate saturation (100 observations). This reduced the sample from 287 to 281 farmworkers. Then 50 farmworkers were removed because they had only one cholinesterase measure, resulting in the final sample size of 231.

Most farmworkers studied were male (90.9%) (Table 1). They ranged in age from 18 to 70 years, with a mean (± SD) of 34.4 ± 10.6 years. More than half (52.0%) had a primary education or less. Most (97.4%) were from Mexico. All spoke Spanish, and 19.1% spoke an indigenous language as a first language. Most had worked in U.S. agriculture before, and 87.9% were migrant workers. More than three-quarters (77.1%) reported having received pesticide safety training at some time. The sample consisted of more than half (62.8%) guestworkers who had H-2A visas.

Cholinesterase levels for the sample varied by month, with the highest level in August and the lowest in June (Table 2). Almost half (48.1%) of workers for whom more than one measurement was available (*n* = 237) had their highest cholinesterase values in August. Depressions of 15% or more from an individual’s maximum cholinesterase activity occurred throughout the season. More than half (50.5%) occurred in June and only 14.3% in August, which is consistent with the trend in mean cholinesterase across the sample. Cholinesterase levels were significantly higher in July than in June, and in August than in all other months (Table 3).

**Figure 1:**
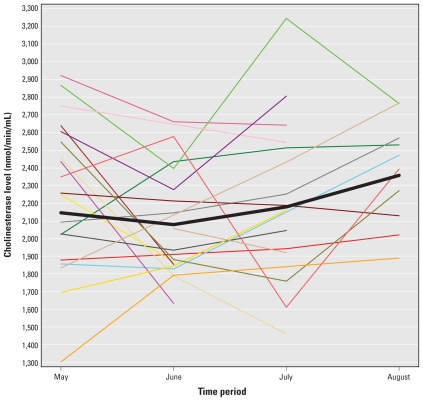
Cholinesterase levels by month for 20 randomly selected farmworkers demonstrates between-person variability in seasonal patterns. The thick black line indicates the sample mean.

Figure 1 shows the study average compared with plots of the cholinesterase levels from 20 randomly selected study participants. These plots indicate substantial variation in patterns of cholinesterase activity among workers during the summer.

In multiple regression adjusted for age, sex, minutes waited to shower, and days worked in the field, reduction from a prior to a later time period was predicted by a greater number of organophosphorus and carbamate pesticides detected in the later time period, as well as the time effects (Table 4). We conducted analyses (data not shown) that included potential predictors of pesticide exposure (e.g., number of days worked in fields, time waited to shower after work). These variables were not significant, so only the most parsimonious model is shown.

## Discussion

These findings indicate a pattern of cholinesterase depression across the agricultural season, with the greatest number of depressions, as well as the lowest mean level of cholinesterase, occurring in June and apparent recovery in July and August. This pattern has face validity, in terms of the local agricultural cycle. Arcury et al. (2009b) reported that approximately 40% of farmworkers worked in vegetable production and fewer than half worked in tobacco in the early to midsummer; by late summer fully 75–80% of farmworkers worked in tobacco. Most insecticides are applied during the spring and early summer and fewer pesticides late in the summer when harvest occurs. In August, when half of the maximums occurred, many of the workers are harvesting tobacco, which should receive no organophosphorus or carbamate pesticide application for several weeks before harvest (Arcury et al. 2009b). The number of organophosphorus and carbamate pesticides detected in urine samples collected at the same time as the cholinesterase samples is associated with cholinesterase depression, providing further evidence that the patterns observed reflect pesticide exposure.

It is likely that the cholinesterase depression detected in the blood samples was attributable solely to inhibition by the exposure to organophosphorus compounds as opposed to the carbamate pesticides. Although both are cholinesterase inhibitors, cholinesterase inhibited by carbamate pesticides will spontaneously reactivate if the sample is extensively diluted or not kept at low temperatures (Nostrandt et al. 1993; Williams and Casterline 1969; Winteringham and Fowler 1966). To elute the dried blood from the filter paper, we had to subject the sample to both extensive dilution and higher temperature (26°C), so we assume that any carbamylated cholinesterase spontaneously reactivated under these conditions. Thus, if there was any cholinesterase inhibition in blood due to carbamate pesticide exposure, that inhibition likely was no longer present by the time we conducted the analysis.

We identified depressions in cholinesterase in this group of farmworkers despite the fact that training in pesticide safety is mandated by U.S. EPA for workers. These findings may indicate that training of these workers is inadequate. Indeed, estimates suggest that one-quarter to two-thirds of farmworkers do not receive training (Arcury et al. 1999; U.S. General Accounting Office 2000; Whalley et al. 2009). Among those who received training, as many as 70% report they do not understand it (Whalley et al. 2009). It is also possible that workers are not following advocated behaviors. For example, both Salvatore et al. (2008) in California and Whalley et al. (2009) in North Carolina reported that 25% and 15% of workers, respectively, wear the same work clothes on ≥ 2 days, which may lead to reexposure to pesticide residues. Other behaviors, such as limited hand washing and failure to wear long sleeves and long pants, may contribute to pesticide exposure. Pesticide exposure also may reflect environmental conditions of farmworkers and not just individual behaviors. For example, farmworker housing is subject to contamination through drift from nearby fields (Quandt et al. 2004; Vallejos et al. 2009). Facilities in a substantial number of farmworker camps have been found not to meet regulations for laundry and bathroom facilities that would allow workers to wash pesticides from clothing and from their skin (Whalley et al. 2009). The cholinesterase data shown here may indicate that protections to minimize exposures are not working and need to be further evaluated and better enforced.

Most other studies of pesticide exposure have used a study design with nonexposed controls (e.g., Cataño et al. 2008; Ntow et al. 2009; Rendón von Osten et al. 2004) or a pre–post design (Gamlin et al. 2007; Thetkathuek et al. 2005) to identify cholinesterase depression. Such designs are not always practical with migrant workers in the eastern United States. Workers frequently move from one crop to the next, so it is not possible to verify when they arrive at a new worksite that they are unexposed. In addition, they work on multiple crops where individual growers make decisions about applying pesticides based on particular field conditions for specific crops, which makes pre–post testing difficult without the cooperation of multiple growers (Arcury et al. 2009a). Also, their often poor-quality housing can expose them to pesticides in nonfield settings (Arcury et al. 2007; Quandt et al. 2004). Thus, this study demonstrates an analytic technique by which multiple cholinesterase measures across a season can be used to identify depressions for individuals. Its advantage is that it does not require a sample with uniform, known exposure or a control group. This approach should be validated in other worker populations.

Measurement of cholinesterase depression is used infrequently in studies of pesticide exposure in U.S. agriculture; the measurement of metabolites for groups of organophosphorus pesticides [e.g., dialkylphosphate pesticides (DAP)] or specific pesticides is more common. However, such studies require knowledge of what pesticides have been applied, and there must be an assay for the pesticide in question. Some organophosphorus pesticides (e.g., acephate, which is used extensively on tobacco) do not affect the common DAP metabolites, and few laboratories have appropriate assays to detect exposure. Therefore, using cholinesterase depression is an efficient way to assess pesticide exposure.

The results of this study should be interpreted in light of several shortcomings. First, data come only from farmworkers in eastern North Carolina; their exposures to pesticides and other environmental factors may differ from those in other regions. Second, because of a lack of a baseline value, a reduction in cholinesterase from any value had to be considered a depression, without regard for time sequence. Recent evidence suggests that as many as 17% of presumed cholinesterase depressions, defined in terms of a 20% change in butyrylcholinesterase, are false positives (Garabrant et al. 2009). Although early evidence suggests that butyrylcholinesterase is more labile than other forms (Sidell and Kaminskis 1975), it is possible that our cut point for defining cholinesterase depression (15% change) may have resulted in misclassification. Third, cholinesterase values are for total cholinesterase; it is not possible to easily divide the cholinesterase into plasma and red blood cell cholinesterase because we used the dried blood spot collection method. Finally, the measure of pesticide metabolites reflects exposure only in the previous few days, whereas cholinesterase reflects cumulative exposure over many weeks. Therefore, additional repeated pesticide exposure measures over many weeks would be necessary to thoroughly characterize pesticide exposure that should be reflected in cholinesterase depressions.

Nonetheless, this study has several strengths. First, we collected data in a relatively large sample with up to four measurement points. This revealed a seasonal pattern of cholinesterase depression, as well as the variability in individual patterns to suggest a variety of exposure sources. The patterns of cholinesterase are related to exposure to organophosphorus and carbamate pesticides, although features of the laboratory analyses point primarily to organophosphorus pesticides.

Despite the development of new pesticides for use in agriculture, organophosphorus pesticides are still the most widely used insecticides. Their health effects are both immediate and long term. Farmworkers are a vulnerable population; because of language barriers and economic pressures, they are frequently not in a position to understand or to request their right to a safe workplace (Arcury and Quandt 2009). Greater monitoring of pesticide exposure and enforcement of existing regulations is needed.

## Figures and Tables

**Table 1:** Personal characteristics of farmworkers, eastern North Carolina, 2007 (*n* = 231).

Characteristic	*n* (%)
Sex	
Male	210 (90.9)
Female	21 (9.1)
Age (years)	
18–24	42 (18.2)
25–29	43 (18.6)
30–39	79 (34.2)
≥ 40	67 (29.0)
Educational attainment (years)	
0–6	120 (52.0)
7–9	81 (35.1)
≥ 10	30 (12.9)
Country of birth	
Mexico	225 (97.4)
United States	1 (0.4)
Other	5 (2.2)
Language spoken	
English	20 (8.7)
Spanish	231 (100.0)
Indigenous language	44 (19.1)
Seasons in U.S. agriculture	
≤ 1	29 (12.7)
2–3	33 (14.5)
4–7	73 (32.0)
≥ 8	93 (40.8)
Ever received pesticide safety training	
No	53 (22.9)
Yes	178 (77.1)
Worker type	
Migrant worker	203 (87.9)
Seasonal worker	28 (12.1)
H-2A visa	
No	86 (37.2)
Yes	145 (62.8)

**Table 2:** Cholinesterase activity (nmol/min/mL), maximum cholinesterase, and cholinesterase depressions, by time period.

Cholinesterase activity	*n*	May	June	July	August
Mean ± SE	231	2145.06 ± 34.3	2079.59 ± 34.2	2181.06 ± 33.6	2357.94 ± 33.6
Maximum [*n* (%)]	231	37 (16.0)	33 (14.3)	50 (21.7)	111 (48.1)
Depression [*n* (%)]	267	73 (39.0)	97 (50.5)	67 (32.5)	30 (14.3)

**Table 3:** Mean pairwise cholinesterase differences (nmol/min/mL), by month.

	May			June			July		
Month	Mean difference	SE	*p*-Value	Mean difference	SE	*p*-Value	Mean difference	SE	*p*-Value
June	65.47	28.0	0.0915	—	—	—	—	—	—
July	−36.00	27.70	0.56	−101.47	27.38	0.0013	—	—	—
August	−212.88	27.75	< 0.0001	−278.35	27.45	< 0.0001	−176.89	26.58	< 0.0001

**Table 4:** Results of multiple regression predicting change in cholinesterase from a prior to a later time period, adjusted for age, sex, minutes waited to shower, and days worked in the fields.

Variable	Regression parameter estimate	SE	*p*-Value
Maximum cholinesterase level	0.032	0.045	0.4748
No. of pesticides detected			
Current month	−29.07	14.25	0.0418
Previous month	12.62	14.55	0.3860
Difference in cholinesterase			
June – May	−42.89	48.75	< 0.0001
July – June	144.80	42.43	< 0.0001
August – July	190.15	41.48	< 0.0001
